# MicroRNA-200a Promotes Phagocytosis of Macrophages and Suppresses Cell Proliferation, Migration, and Invasion in Nasopharyngeal Carcinoma by Targeting CD47

**DOI:** 10.1155/2020/3723781

**Published:** 2020-02-20

**Authors:** Yunteng Zhao, Xiaoxiao Yu, Haocheng Tang, Ri Han, Xianwen Wang, Jianqi Wang, Ke Wang, Gang Li

**Affiliations:** ^1^Department of Otolaryngology-Head and Neck Surgery, Nanfang Hospital, Southern Medical University, Guangzhou, China; ^2^Department of Otolaryngology-Head and Neck Surgery, The Third Affiliated Hospital of Southern Medical University, Southern Medical University, Guangzhou, China

## Abstract

Nasopharyngeal carcinoma (NPC) causes severe oncogenic lesions in the nasopharynx. CD47, a transmembrane integrin-associated protein, plays a key role in the ability of tumor cells to escape phagocytosis, working as an immune checkpoint in the immune response. Besides this role, CD47 has been reported to regulate cell proliferation and migration. The present study addresses the relationship between CD47 and microRNA-200a and examines their regulatory mechanisms in NPC. Bioinformatics analyses and dual-luciferase reporter assays were used to confirm the putative relationship between miR-200a and CD47, and their interaction was further detected using western blotting and RT-PCR. Further, results showed that miR-200a affect NPC cell proliferation, migration, and invasion by regulating CD47. A cell phagocytosis assay showed that miR-200a and a CD47 monoclonal antibody increased the sensitivity of NPC cells to macrophage phagocytosis by inhibiting the functions of CD47. Additionally, miR-200a expression was suppressed and CD47 expression increased in both clinical NPC tissues and cell lines. Taken together, these results show the miR-200a/CD47 combination as a potential therapeutic for treatment of NPC.

## 1. Introduction

Nasopharyngeal carcinoma (NPC) affects the nasopharynx and varies in prevalence by geographic region and ethnicity [1]. Approximately 80,000 new NPC cases are annually diagnosed worldwide, resulting in 50,000 cancer-related deaths [2]. Patients with NPC exhibit extreme suppression of the immune response, and studies have reported that immunosurveillance mechanisms are associated with NPC progression [3]. Although current radiotherapy- and chemotherapy-based comprehensive strategies utilizing intensity-modulated radiotherapy have shown great progress for the treatment of NPC, the 5-year survival rate remains at approximately 70% [4], with 15-58% of affected patients experiencing local tumor recurrence or metastasis [5]. Therefore, as with other life-threatening cancers, the development of more efficient therapeutic strategies is needed.

Accumulating evidence suggests that cancer cells can impair the immune system and evade phagocytosis by macrophages via the activation of CD47 signaling [6, 7]. Indeed, CD47 is considered a fundamental “do not eat me” signal [8–11], and it negatively regulates phagocytosis by binding to signal regulatory protein alpha (SIRP*α*) on phagocytic cells [12, 13]. The blockade of CD47-SIRP*α* interactions has been shown to enhance the phagocytic activity of phagocytes, such as macrophages, toward tumor cells, thereby resulting in the efficient eradication of tumor cells [14]. In addition, CD47 blockade also stimulates cytotoxic T cell function by macrophages or dendritic cells, thereby providing another potential benefit for CD47-based therapy. Hence, targeting the CD47-SIRP*α* signaling system is a promising strategy for cancer treatment, including NPC.

MicroRNAs (miRNAs) are small, noncoding RNAs containing 20-25 nucleotides in length. By binding to complementary sequences in the 3′ untranslated regions (UTRs), miRNAs negatively regulate the expression of genes and participate in multiple biological processes [15]. Previous studies have demonstrated that miRNAs can precisely modulate immune networks by regulating key genes that affect the immune system. It has been reported that CD47 expression in multiple tumors is regulated by microRNAs (miRNAs) including miR-133a, miR-155, and miR-708 [16–18]. Moreover, bioinformatics analysis has suggested that miR-200a might target CD47. MicroRNA-200a (miR-200a) acts as a tumor suppressor in various cancers, including NPC, and is an important factor in the miR-200 family, which includes five members: miR-200a, miR-200b, miR-200c, miR-141, and miR-429 [19–21]. A recent study demonstrated a potential interaction between miR-200a and PD-L1, showing that miR-200 family members inhibit PD-1 signaling by targeting PD-L1 to prevent tumors from escaping immune surveillance [22]. Therefore, it would be interesting to determine whether miR-200a targets CD47 in parallel to PD-L1 to exert regulatory effects on immune checkpoints in cancer. Based on recent reports that miRNAs efficiently regulate immune responses as modulators of immune checkpoint molecules and their potential as cancer therapeutic targets and agents [17, 23, 24], it is reasonable to speculate that miRNAs could affect CD47 and exert associated effects on immune checkpoints during NPC tumorigenesis.

## 2. Materials and Methods

### 2.1. Patients and NPC Specimen Collection

NPC biopsy specimens (*n* = 40) and noncancerous nasopharyngitis biopsy samples (*n* = 20) were collected from Nanfang Hospital (Southern Medical University, Guangzhou, China). All donors provided signed informed written consent. The experimental protocol was approved by the ethics committee of Nanfang Hospital. All of the procedures were performed in accordance with the Declaration of Helsinki and relevant policies in China.

### 2.2. Cell Culture and Transfection

The human NPC 5-8F, 6-10B, SUNE1, CNE1, and CNE2 cell lines were cultured in RPMI-1640. Human nasopharyngeal epithelial cells (NP69 cells) were cultured in a serum-free medium (keratinocyte-SFM) (Thermo Fisher Scientific, Gibco, USA). HEK 293 T cells were cultured in DMEM/High Glucose (Thermo Fisher Scientific, Gibco, USA). All cell lines were incubated in media supplemented with 100 U/ml penicillin, 100 U/ml streptomycin, and 10% fetal bovine serum (FBS, BI, Germany) at 37°C in a humid atmosphere containing 5% CO_2_. All cell lines were obtained from the Laboratory of Head and Neck Oncology, Nanfang Hospital, Southern Medical University (Guangzhou, China), and the protocols, including the use of the cell lines, were approved by the ethics committee of Nanfang Hospital.

For cell transfection, the following groups were set up: (a) MOCK (blank control), (b) miR-NC (scrambled sequence, abbreviated as NC), (c) miR-200a mimic (artificially synthesized miR-200a analog), (d) miR-200a inhibitor (artificially synthesized miRNA which can specifically inhibit endogenous miR-200a activity), (e) CD47 siRNA, and (f) miR-200a inhibitor+CD47 siRNA groups. All RNA oligonucleotides were provided by RiboBio Company (Guangzhou, China). Transfection of NPC cells was carried out with Lipofectamine 3000 (Thermo Fisher Scientific, Invitrogen, USA), and transfected at a final concentration of 150 nM for all RNA oligonucleotides in CNE1 and CNE2 cells in accordance with the manufacturer's instructions. Transfection efficiencies of CNE1 and CNE2 cell lines are 90 ± 7% and 86 ± 9%, respectively.

### 2.3. In Situ Hybridization (ISH)

Specimens were fixed in formalin and embedded in paraffin. An ISH assessment of miR-200a levels was performed with a miRCURY LNA™ microRNA ISH Optimization Kit (Exiqon, Denmark) according to the manufacturer's instructions. Data were analyzed under a microscope (BX51 Olympus, Japan).

### 2.4. Immunohistochemistry (IHC)

For IHC assays, NPC specimens were incubated at 60°C for 2 h before treatment with dimethyl benzene for dewaxing. The sections were then hydrated in a gradient of alcohol and washed with ddH_2_O. Subsequently, they were fixed with 3% H_2_O_2_ for 15 min and washed with PBS three times at room temperature. After incubation with primary antibodies against CD47 (1 : 100, Santa Cruz, Dallas, TX, USA) at 37°C for 30 min and 4°C overnight, the corresponding secondary antibody (Santa Cruz, Dallas, TX, USA) was added for 30 min at 37°C. Next, the sections were sequentially incubated with HRP-labeled avidin for 30 min at 37°C and DAB for 3-10 min, and the reaction was stopped with ddH_2_O. Counterstaining was performed with hematoxylin. The sections were finally analyzed under a microscope (BX51, Olympus, Japan).

The percentage of stained cells was evaluated by the Formwitz method and scored as follows: 1 (up to 25% positive cells), 2 (25% to 50% positive cells), 3 (50% to 75% positive cells), and 4 (more than 75% positive cells). Intensity scores ranged from 0 to 3: 0, no staining; 1, weak staining; 2, moderate staining; and 3, strong staining. The product of both subscores yielded a final score ranging from 0 to 12.

### 2.5. Dual-Luciferase Assay

For the dual-luciferase assay, wild-type (WT) CD47 3′UTR was amplified by PCR and ligated into the psiCHECK-2 plasmid (XhoI site) with One-Step Cloning Kit (Vazyme, Nanjing, China). Then, the sequence of the WT CD47 promoter was mutated by homologous recombination (5′-GTCACAA-3′) and ligated into the psiCHECK-2 plasmid (XhoI site) with One-Step Cloning Kit (Vazyme, Nanjing, China). Transfection of different combinations of miR-NC, miR-200a mimic, psiCHECK-2 plasmid, WT CD47 3′UTR, and mutant CD47 3′UTR was performed with Lipofectamine™ 3000 according to the manufacturer's instructions. Luciferase activity was detected with a Dual-Luciferase Assay Kit (Promega, Madison, WI, USA) according to the manufacturer's instructions. Cells were cotransfected with 1 *μ*g of WT or mutant reporter plasmids and 150 nM miR-200a mimic with Lipofectamine 3000. Cells were harvested and lysed at 24 h after transfection. Firefly and Renilla luciferase activities were read on a microplate reader (GloMax, Promega, Madison, WI, USA).

### 2.6. Real-Time PCR (RT-PCR)

Total RNA was extracted with RNAiso Plus (Takara, Japan) according to the manufacturer's instructions. Mature miR-200a and the endogenous control U6 were assessed with an All-in-One™ miRNA Synthesis Kit (GeneCopoeia, USA). To analyze the CD47 and GAPDH (endogenous control) expression levels, we carried out reverse transcription and qPCR with PrimeScript™ RT Master Mix Kit (Takara, Japan) and SYBR® Premix Ex Taq™ Kit (Takara, Japan), respectively. CD47 expression was quantified relative to GAPDH levels. The following primers were used: CD47, forward 5′-TCCGGTGGTATGGATGAGAAA-3′ and reverse 5′-ACCAAGGCCAGTAGCATTCTT-3′; GAPDH, forward 5′-ACAACTTTGGTATCGTGGAAGG-3′ and reverse 5′-GCCATCACGCCACAGTTTC-3′. Fold change differences in miRNA or mRNA expression were assessed with a real-time PCR detection system (LightCycler 480, Roche, CH) by the 2^−*ΔΔ*Ct^ method.

### 2.7. Western Blotting

Cells were lysed in RIPA buffer (Beyotime, Shanghai, China) supplemented with protease inhibitors (Beyotime, Shanghai, China). Total protein was quantified with a BCA Protein Assay Kit (Thermo Scientific, USA). Equal amounts of protein were separated by 10% SDS-PAGE and electrophoretically transferred onto PVDF membranes (Millipore, Billerica, MA, USA). After blocking with 5% nonfat milk, the membranes were incubated with anti-CD47 (B6H12.2, Abcam, Cambridge, UK) and anti-GAPDH (Santa Cruz, Dallas, TX, USA) antibodies at 4°C overnight and further incubated with matched HRP-conjugated rabbit or mouse secondary antibodies (Abcam, Cambridge, UK). Proteins were detected by enhanced chemiluminescence (Tanon, Shanghai, China).

### 2.8. Cell Proliferation Assay

The ability of NPC cells to proliferate was measured by EdU assays (Cell-Light EdU Apollo 567 In Vitro Imaging Kit, RiboBio, Guangzhou, China). After transfection with RNA oligonucleotides, CNE1 and CNE2 cells were incubated with 10 *μ*M EdU for 2 h and fixed with 4% paraformaldehyde for 30 min at room temperature. Then, the cells were incubated with 2 mg/ml glycine for 5 min followed by permeabilization with 0.25% Triton X-100 for 10 min and EdU staining for 30 min. EdU-positive cells were counted under a confocal laser-scanning microscope (Olympus FV1000, Japan) in five random fields.

### 2.9. Cell Colony Formation Assay

For the colony formation assay, cells were suspended in 10% serum-containing medium and inoculated into plates at a density of 200 cells per plate. After one week of culture, colonies containing more than 50 cells were stained with crystal violet.

### 2.10. Transwell Assay

Transwell experiments were performed to evaluate the migratory and invasive abilities of NPC cells. After transfection, 1 × 10^4^ cells were seeded into the upper chambers of Transwell plates (Corning, NY, USA). RPMI-1640 containing 10% FBS was added to the lower chambers. The cells were incubated at 37°C in a humidified atmosphere with 5% CO_2_ for 24 h. Upon culture, the membranes were fixed with 4% paraformaldehyde for 20 min. Then, the membranes were stained with crystal violet for 5 min, and adherent cells were counted. The invasive ability of NPC cells was measured as described above using polycarbonate membranes previously coated with 40 *μ*l Matrigel (1.5 mg/ml; BD Biosciences, San Jose, CA, USA) at 37°C for 2 h to form a reconstituted basement membrane.

### 2.11. Phagocytosis Assay

Monocytes (human peripheral blood-derived mononuclear cells) were obtained from healthy donors after informed consent (LDEBIO, Guangzhou, China). Monocyte-derived macrophage cultures were established in RPMI 1640 supplemented with 10% FBS and 50 ng/ml M-CSF (eBioscience, USA) for 7 days.

For in vitro phagocytosis analysis, 1 × 10^4^ monocyte-derived macrophages were plated in each well of 48-well tissue culture plates and labeled with a PKH67 Green Fluorescent Cell Linker Kit (Sigma-Aldrich, St. Louis, MO, USA) according to the manufacturer's instructions. Macrophages were incubated in serum-free medium for 2 h followed by an addition of 1 × 10^4^ PKH26-labeled NPC cells. Anti-CD47 (B6H12, 10 mg/ml, Abcam, Cambridge, UK) and IgG1 control antibodies, respectively, were then added, and cells were incubated for 3 h at 37°C. After washing in PBS, the cells were fixed with 0.4% paraformaldehyde. The phagocytic index was assessed under a fluorescence microscope (lx71, Olympus, Japan) as the number of red phagocytosed NPC cells per 100 green macrophages in randomly selected microscopic fields.

### 2.12. Statistical Analysis

Data are presented as the mean ± SD. Nonnormally distributed data were analyzed by the Mann–Whitney *U* test. The correlation between miR-200a and CD47 expression in clinical NPC samples was assessed by Spearman's correlation analysis. Normally distributed data were analyzed by Student's *t*-tests. Two-tailed *P* < 0.05 was considered statistically significant. All statistical analyses were performed with SPSS version 22.0 (IBM, Armonk, NY, USA).

## 3. Results

### 3.1. miR-200a Is Downregulated and CD47 Is Upregulated in NPC

**Figure 1:**
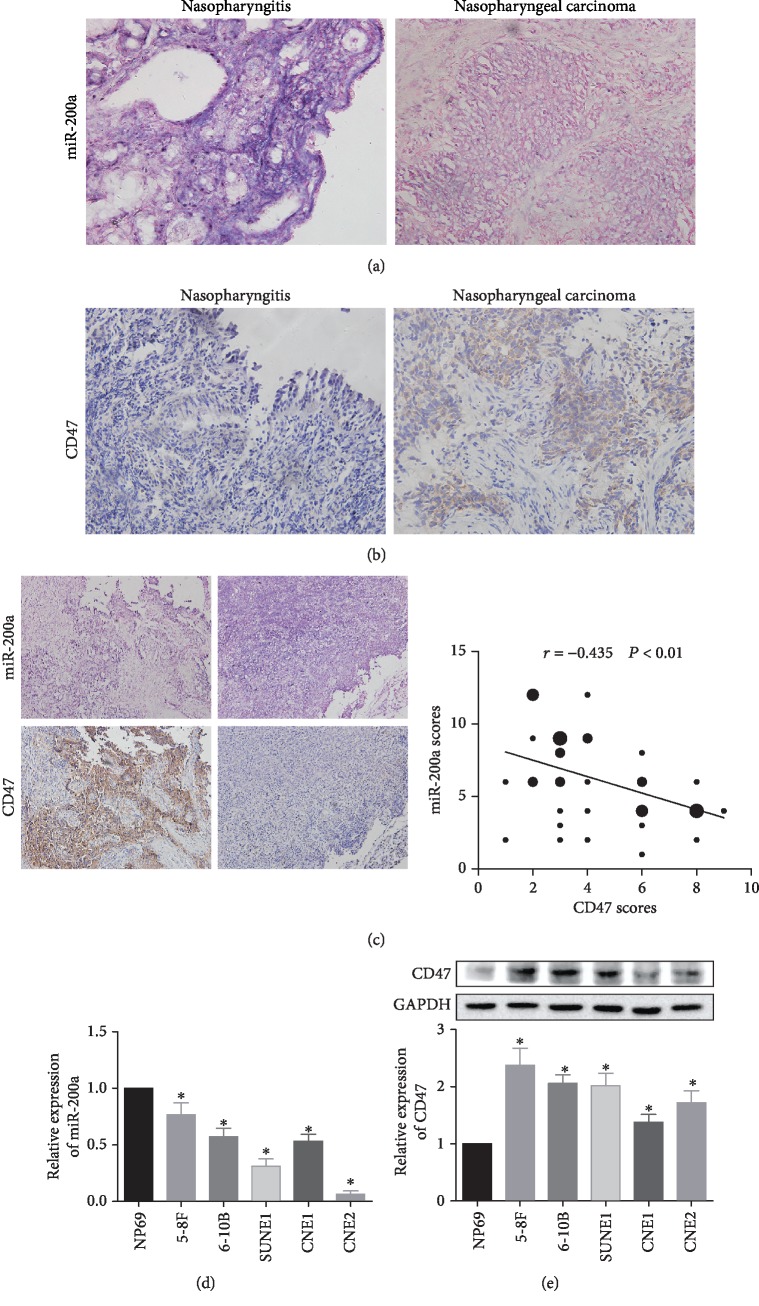
Expression levels of miR-200a and CD47 in NPC. Notes: the expression of miR-200a was lower (a) and that of CD47 (b) was higher in NPC tissues than in nasopharyngitis samples. (c) Based on Spearman's correlation analysis, miR-200a expression was negatively correlated with CD47 levels. The expression of miR-200a was lower (d) while that of CD47 was higher (e) in NPC cells than in NP69 cells. ^∗^*P* < 0.05 vs. the NP69 group. Abbreviations: CD47: cluster of differentiation 47; NC: negative control.

The levels of miR-200a were detected by ISH in NPC patients, with normal nasopharyngitis tissues used as controls. As shown in Figure 1(a) and Table 1, miR-200a levels in the nucleus and cytoplasm were higher (stained blue) in nasopharyngitis tissues than in NPC tissues. Meanwhile, the amount of CD47 protein in NPC samples was determined by IHC. CD47 expression was higher in NPC samples than in nasopharyngitis tissues (Figure 1(b) and Table 2). Furthermore, with NPC progression, expression of miR-200a decreased (Table 3) and that of CD47 increased. Based on Spearman's correlation analysis, miR-200a expression was negatively correlated with CD47 levels (Figure 1(c)) (*r* = −0.435, *P* < 0.01).

In addition to determining the levels of these two molecules in clinical specimens, we measured their amounts in NPC cell lines. The expression levels of miR-200a were significantly lower in various NPC cell lines, including 5-8F, 6-10B, SUNE1, CNE1, and CNE2, than in nontumorigenic nasopharyngeal epithelial cells (NP69 cells) (Figure 1(d)). Among these cell lines, 5-8F, 6-10B, and CNE1 had the highest miR-200a expression levels.

Moreover, CD47 protein levels were evaluated in the above NPC cell lines by western blotting. The results show that the CD47 were significantly higher in NPC cells than in NP69 cells (Figure 1(e)).

### 3.2. miR-200a Regulates CD47 Expression by Directly Targeting the 3′UTR of the CD47 mRNA

**Figure 2:**
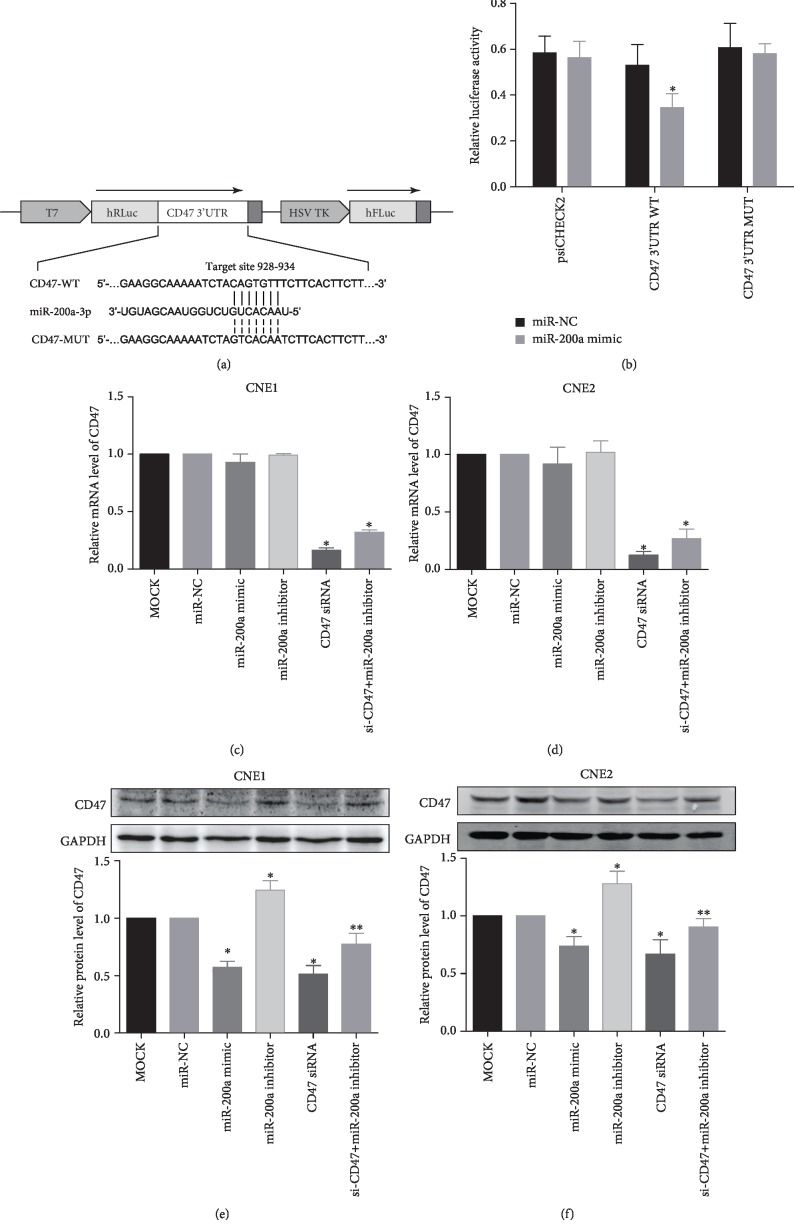
miR-200a directly regulates CD47 expression in NPC cells. Notes: (a, b) based on a dual-luciferase assay, miR-200a was predicted to directly bind to the 3′UTR of CD47 mRNA, and cotransfection of the miR-200a mimic and wild-type promoter resulted in decreased luciferase activity. (c, d) At the mRNA level, CD47 expression was inhibited by the CD47 siRNA in both cell lines. (e, f) At the protein level, CD47 expression was inhibited by the miR-200a mimic in both cell lines. ^∗^*P* < 0.05 vs. the miR-NC group. ^∗∗^*P* < 0.05 vs. the miR-200a inhibitor group. Abbreviations: UTR: untranslated regions; WT: wild type; MUT: mutant-type; CD47: cluster of differentiation 47.

TargetScan prediction database (http://www.targetscan.org/) revealed CD47 as a potential target of miR-200a (Figure 2(a)). To verify this finding, we performed dual-luciferase assays. The results show that cotransfection of miR-200a mimic and psiCHECK-2-wt CD47 3′UTR (CD47 WT) resulted in significantly lower luciferase activity than cotransfection of miR-200a mimic with psiCHECK-2 (control) or psiCHECK-2-mut CD47 3′UTR (CD47 MUT) (Figure 2(b)). In addition, luciferase activity decreased markedly in the CD47 WT group transfected with miR-200a mimic. Furthermore, different combinations of miR-200a mimic, miR-200a inhibitor, and siRNA targeting CD47 were transfected into CNE1 (well differentiated) and CNE2 (poorly differentiated) cells, and the expression levels of miR-200a and CD47 were evaluated. The mRNA level of CD47 was analyzed by qRT-PCR in CNE1 and CNE2 cells that were transfected with miR-200a. No significant difference was found between miR-200a-transfected and NC-transfected cells (Figures 2(c) and 2(d)). Nevertheless, the protein expression of CD47 was significantly suppressed by CD47 siRNA, and the overexpression of miR-200a also inhibited CD47 expression (Figures 2(e) and 2(f)). Cotransfection of the miR-200a inhibitor restored CD47 protein expression in cells transfected with CD47 siRNA (Figures 2(e) and 2(f)). Transfection of miR-200a did not alter the expression of CD47 mRNA, but it inhibited the expression of CD47 similar to siRNA CD47, indicating that miR-200a can inhibit protein translation and decrease the expression of CD47 by binding with CD47 mRNA. Taken together, these results indicate that miR-200a inhibited the expression of CD47 by directly binding to the 3′UTR of its mRNA.

### 3.3. miR-200a Suppresses NPC Cell Proliferation and Colony Formation by Downregulating CD47

**Figure 3:**
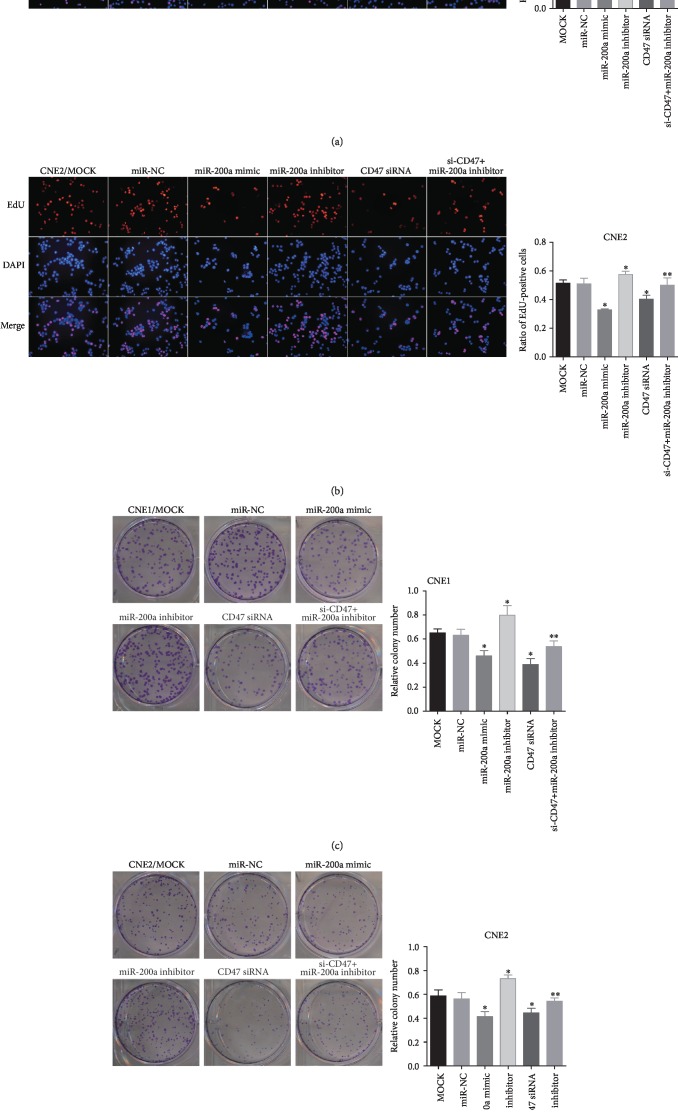
Proliferation and colony formation in CNE1 and CNE2 cells. Notes: (a, b) transfection of miR-200a and CD47 siRNA suppressed proliferation and (c, d) colony formation. Cotransfection with CD47 siRNA and miR-200a inhibitor rescued the effects of CD47 knockdown in CNE1 and CNE2 cells. ^∗^*P* < 0.05 vs. the miR-NC group. ^∗∗^*P* < 0.05 vs. the miR-200a inhibitor group. Abbreviations: CD47: cluster of differentiation 47; EdU: 5-ethynyl-2′-deoxyuridine; DAPI: 4′,6-diamidino-2-phenylindole; NC: negative control.

To determine the effects of miR-200a/CD47 on tumorigenesis in NPC, we examined proliferation and colony formation in various NPC cell lines. After CNE1 and CNE2 cells were transfected with miRNA constructs, proliferation in various groups (miR-200a mimic, NC, miR-200a inhibitor, CD47 siRNA, miR-200a inhibitor+CD47 siRNA, and MOCK groups) was detected by EdU assays. As shown in Figures 3(a) and 3(b), significantly fewer proliferating cells were observed in the CD47 siRNA and miR-200a mimic groups than in the MOCK and NC groups (*P* < 0.05). However, there were more proliferating cells in the miR-200a inhibitor group than in the other groups (*P* < 0.05). The number of proliferating cells in the miR-200a inhibitor+CD47 siRNA group was significantly lower than that in the miR-200a inhibitor group but higher than that in the CD47 siRNA group (both *P* < 0.05). These findings indicated that CD47 siRNA reversed the enhancement in cell proliferation induced by the miR-200a inhibitor (*P* < 0.05).

To assess the effect of miR-200a on colony formation in NPC cells, we cultured CNE1 and CNE2 cells for 14 days to allow colony formation (Figures 3(c) and 3(d)). The number of colonies was lower in cells transfected with miR-200a mimic and CD47 siRNA and higher in cells transfected with the miR-200a inhibitor than in cells transfected with their respective controls. Additionally, the miR-200a inhibitor+CD47 siRNA group had significantly fewer colonies than the miR-200a inhibitor group but more colonies than in the CD47 siRNA group (both *P* < 0.05), indicating that CD47 siRNA reversed the enhancement in cell proliferation induced by the miR-200a inhibitor (*P* < 0.05). These data suggested that miR-200a regulated proliferation and colony formation by suppressing CD47 signaling in NPC cells.

### 3.4. miR-200a Suppresses NPC Cell Migration and Invasion by Downregulating CD47

**Figure 4:**
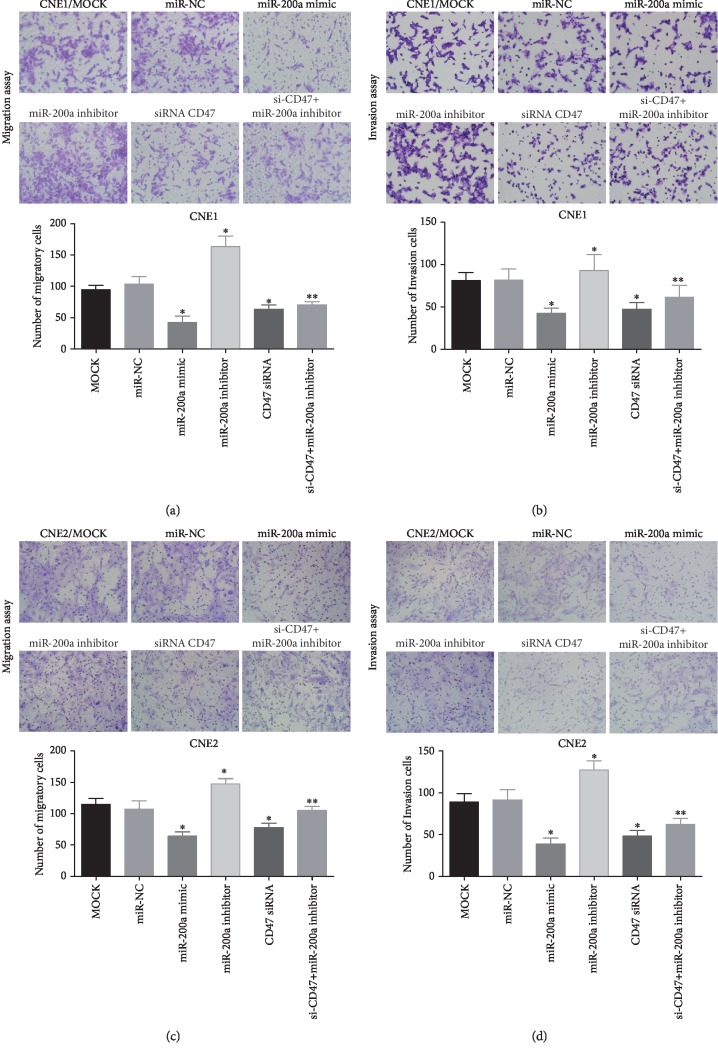
Migration and invasion of CNE1 and CNE2 cells. Notes: transfection with miR-200a and CD47 siRNA inhibited migration (a, c) and invasion (b, d) in CNE1 and CNE2 cells. Cotransfection with CD47 siRNA and miR-200a inhibitor rescued the effect of CD47 knockdown on the metastatic potential of CNE1 and CNE2 cells. ^∗^*P* < 0.05 vs. the miR-NC group. ^∗∗^*P* < 0.05 vs. the miR-200a inhibitor group. Abbreviations: CD47: cluster of differentiation 47; NC: negative control.

To determine the effect of miR-200a on the migration and invasion of NPC cells, miR-200a mimic, miR-200a inhibitor, and CD47 siRNA were transfected into CNE1 and CNE2 cells. The effects of these RNA oligonucleotides on NPC cell migration and invasion were evaluated via Transwell assays (Figure 4) or wound healing assays ([Supplementary-material supplementary-material-1]). The results show that the invasive and migratory abilities of CNE1 (Figures 4(a) and 4(b)) and CNE2 (Figures 4(c) and 4(d)) cells were suppressed after transfection with miR-200a mimic (*P* < 0.05). Conversely, cells transfected with the miR-200a inhibitor exhibited increased invasiveness and migratory abilities. In addition, downregulation of CD47 in NPC by the RNAi technology clearly affected cell invasion and migration, and the effects were similar to those observed for miR-200a mimic. To assess the possibility that miR-200a acts on NPC cells by inhibiting CD47 signaling, we cotransfected CNE1 and CNE2 cells with CD47 siRNA and a miR-200a inhibitor. The results show that impairments in migration and invasion observed in NPC cells as a result of CD47 suppression were rescued by the miR-200a inhibitor (Figure 4). Given that miR-200a directly binds to the 3′UTR of the CD47 gene, we conclude that miR-200a antagonized NPC cell invasion and migration by suppressing CD47 signaling.

### 3.5. Anti-CD47 Antibody and miR-200a Promotes NPC Cell Phagocytosis by Macrophages

**Figure 5:**
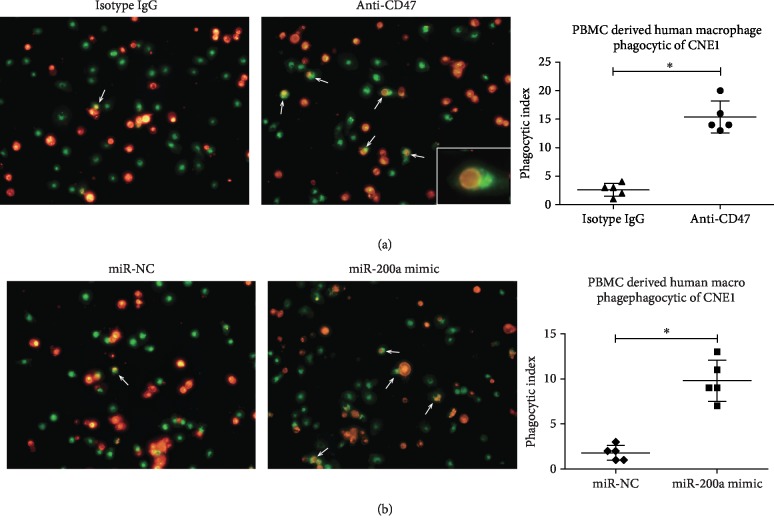
Anti-CD47 antibody and miR-200a promote phagocytosis of NPC cells by human macrophages. Notes: (a) human peripheral blood- (PB-) derived macrophages (green) are shown phagocytosing NPC cells (red) in the presence of a blocking anti-CD47 monoclonal antibody (B6H12) or IgG1 isotype control antibody. (b) Phagocytosed NPC cells (red) are shown in the miR-200a mimic and miR-NC groups. ^∗^*P* < 0.01. Abbreviations: CD47: cluster of differentiation 47; PBMC: peripheral blood mononuclear cell; IgG: immunoglobulin G.

Regarding the immune checkpoint-related mechanism by which miR-200a promotes NPC, we hypothesized that miR-200a acts via CD47 regulation, which might affect NPC cell phagocytosis by macrophages. To test this hypothesis, we conducted phagocytosis assays. Indeed, cells incubated with anti-CD47 monoclonal antibodies were more sensitive to phagocytosis by macrophages than CNE1 cells treated with the isotype control IgG. CNE1 cells and macrophages were stained red and green, respectively, with more phagocytosed NPCs found in the anti-CD47 antibody- (B6H12-) treated group (*P* < 0.01) (Figure 5(a)).

We next evaluated the effect of specific miR-200a mimic on CD47 inhibition. Similar to the results of anti-CD47 antibody treatment, inhibition of CD47 with miR-200a increased the number of NPC cells phagocytosed by macrophages (Figure 5(b)) (*P* < 0.01). Although the effect of miR-200a mimic was weaker than that of anti-CD47 antibody, the results still indicated that the miR-200a/CD47 interaction plays a key role in the immune evasion of NPC cells.

## 4. Discussion

The ability to block immune checkpoints represents one of the most important advances in anticancer therapy developed in the past decade. CD47, a new immune checkpoint, is overexpressed in almost all malignant human tumors [6, 8, 10, 25]. In our study, we found that the miR-200a/CD47 cascade was partially involved in the immune escape process of NPC, presenting the miR-200a/CD47 cascade as a novel target for NPC treatment.

miRNAs posttranscriptionally modulate gene expression by binding to the 3′UTRs of their target genes. Xu et al. found that miR-424 activates cytotoxic T cells in ovarian cancer by blocking the immune checkpoint PD-L1 in tumor cells, resulting in immune activation [26]. Whether microRNAs affect the antitumor action of the immune system against NPC by regulating CD47 has not been evaluated clearly. First, we found that the miR-200a expression was downregulated and the CD47 expression was upregulated in NPC specimens. A correlation analysis showed that in clinical samples, the expression level of miR-200a was negatively correlated with CD47 levels. This result supported the conclusions from a bioinformatics analysis that showed that CD47 was a potential target of miR-200a. To further confirm a direct interaction between these two molecules, we performed a dual-luciferase assay. The data showed that miR-200a directly binds to the 3′UTR of the CD47 gene.

The roles of miR-200a and CD47 in NPC progression were further assessed in CNE1 and CNE2 cells. We found that miR-200a suppressed proliferation, invasion, and migration in NPC cells, while miR-200a inhibition had the opposite effects, thus corroborating the data from Xia et al. [27]. These results support the promising anti-NPC potential of miR-200a. CD47 inhibition had a similar effect on NPC cells as miR-200a mimic. Furthermore, NPC cells cotransfected with miR-200a inhibitor and CD47 siRNA, rescued the cells from impairments caused by miR-200a knockdown, thus providing partial support for our hypothesis that the anti-NPC function of miR-200a is partly dependent on CD47 suppression. Previous studies have demonstrated that the inhibition of cell proliferation and colony formation by CD47 knockdown can be attributed to the suppression of cancer stem cell character [28]. Based on our findings, we suspect that the effects on cell proliferation and migration might represent cell autonomous activity that is independent of the immune system.

Another major purpose of the current study was to assess NPC cell phagocytosis by macrophages. The interaction between CD47 and macrophages was blocked by the anti-CD47 antibody. Anti-CD47 antibody administration resulted in a dramatically higher proportion of NPC cells phagocytosed by macrophages than did treatment with control IgG, thus confirming that CD47 plays a key role in tumor cell escape from immune surveillance [29, 30]. Moreover, miR-200a mimic achieved a similar effect on phagocytosis by increasing the number of NPC cells phagocytosed by macrophages. Therefore, decreasing CD47 expression with either monoclonal antibodies or miR-200a mimic might represent a promising strategy to increase the immune sensitivity of NPC cells. Preliminary trials of an anti-CD47 monoclonal antibody have achieved some initial success [31]. However, there are still challenges, including unstable therapeutic effects and difficulty in controlling side effects.

## 5. Conclusions

In our study, we demonstrate for the first time that miR-200a promotes NPC cell phagocytosis by downregulating CD47, preventing immune escape. In addition, miR-200a acts as a tumor suppressor by inhibiting proliferation and metastasis in NPC [27]. Combining immunotherapeutic drugs that target different aspects of the same mechanism can often result in a significant increase in efficacy. Interestingly, miR-200a blocks CD47 at the posttranscriptional level and may act synergistically with anti-CD47 antibodies to affect protein levels, which could further improve its efficacy and reduce complications. In summary, this study provides preliminary data support for the use of miR-200a/CD47 as a therapeutic target in NPC. Additional efforts are needed to evaluate the precise mechanism involved in the miR-200a/CD47 cascade in NPC.

## Figures and Tables

**Table 1:** Expression of miR-200a in nasopharyngeal carcinoma and nasopharyngitis tissues.

	miR-200a ISH				*P*
	-	+	++	+++	
NPC (40)	0	18	12	10	0.002
Nasopharyngitis (20)	0	2	6	12	

**Table 2:** Expression of CD47 in nasopharyngeal carcinoma and nasopharyngitis tissues.

	CD47 IHC				*P*
	-	+	++	+++	
NPC (40)	0	25	11	4	0.0003
Nasopharyngitis (20)	9	8	3	0	

**Table 3:** Expression of miR-200a in nasopharyngeal carcinoma tissues of different TNM stages.

	miR-200a ISH				*P*
	-	+	++	+++	
T1-T2 (18)	0	3	9	6	0.017
T3-T4 (22)	0	14	4	4	
N0-N1 (9)	0	1	4	4	0.044
N2-N3 (31)	0	16	9	6	

## Data Availability

The data used to support the findings of this study are available from the corresponding author upon request.

## Supplementary Materials

Supplementary Materials Supplemental Figure 1: migration of CNE1 and CNE2 cells by wound healing assay. (a) miR-200a suppresses CNE1 cell migration by downregulating CD47. (b) miR-200a suppresses CNE2 cell migration by downregulating CD47. ^∗^*P* < 0.05 vs. the miR-NC group. ^∗∗^*P* < 0.05 vs. the miR-200a inhibitor group.

## References

[1] Chua M. L. K., Wee J. T. S., Hui E. P., Chan A. T. C. (2016). Nasopharyngeal carcinoma. *Lancet*.

[2] Petersson F. (2015). Nasopharyngeal carcinoma: a review. *Seminars in Diagnostic Pathology*.

[3] Jiang F., Yu W., Zeng F. (2019). PD-1 high expression predicts lower local disease control in stage IV M0 nasopharyngeal carcinoma. *BMC Cancer*.

[4] Yoshizaki T., Ito M., Murono S., Wakisaka N., Kondo S., Endo K. (2012). Current understanding and management of nasopharyngeal carcinoma. *Auris Nasus Larynx*.

[5] Strazzulla A., Barreca G. S., Giancotti A. (2015). Nasopharyngeal carcinoma: review of the literature with a focus on therapeutical implications. *Le Infezioni in Medicina*.

[6] Yoshida K., Tsujimoto H., Matsumura K. (2015). CD47 is an adverse prognostic factor and a therapeutic target in gastric cancer. *Cancer Medicine*.

[7] Jaiswal S., Chao M. P., Majeti R., Weissman I. L. (2010). Macrophages as mediators of tumor immunosurveillance. *Trends in Immunology*.

[8] Majeti R., Chao M. P., Alizadeh A. A. (2009). CD47 is an adverse prognostic factor and therapeutic antibody target on human acute myeloid leukemia stem cells. *Cell*.

[9] Hofner T., Macher-Goeppinger S., Klein C. (2014). Expression and prognostic significance of cancer stem cell markers CD24 and CD44 in urothelial bladder cancer xenografts and patients undergoing radical cystectomy. *Urologic Oncology*.

[10] Kaur S., Elkahloun A. G., Singh S. P. (2016). A function-blocking CD47 antibody suppresses stem cell and EGF signaling in triple-negative breast cancer. *Oncotarget*.

[11] Li F., Lv B., Liu Y. (2017). Blocking the CD47-SIRP*α* axis by delivery of anti-CD47 antibody induces antitumor effects in glioma and glioma stem cells. *Oncoimmunology*.

[12] Oldenborg P. A., Gresham H. D., Lindberg F. P. (2001). CD47-signal regulatory protein *α* (Sirp*α*) regulates Fcgamma and complement receptor-mediated phagocytosis. *The Journal of Experimental Medicine*.

[13] Weiskopf K. (2017). Cancer immunotherapy targeting the CD47/SIRP*α* axis. *European Journal of Cancer*.

[14] Veillette A., Chen J. (2018). SIRP*α*-CD47 immune checkpoint blockade in anticancer therapy. *Trends in Immunology*.

[15] Bartel D. P. (2004). MicroRNAs: genomics, biogenesis, mechanism, and function. *Cell*.

[16] Huang W., Wang W. T., Fang K. (2018). MIR-708 promotes phagocytosis to eradicate T-ALL cells by targeting CD47. *Molecular Cancer*.

[17] Rothchild A. C., Sissons J. R., Shafiani S. (2016). MiR-155-regulated molecular network orchestrates cell fate in the innate and adaptive immune response to Mycobacterium tuberculosis. *Proceedings of the National Academy of Sciences of the United States of America*.

[18] Suzuki S., Yokobori T., Tanaka N. (2012). CD47 expression regulated by the miR-133a tumor suppressor is a novel prognostic marker in esophageal squamous cell carcinoma. *Oncology Reports*.

[19] O'Brien S. J., Carter J. V., Burton J. F. (2018). The role of the miR-200 family in epithelial-mesenchymal transition in colorectal cancer: a systematic review. *International Journal of Cancer*.

[20] Jurmeister S., Baumann M., Balwierz A. (2012). MicroRNA-200c represses migration and invasion of breast cancer cells by targeting actin-regulatory proteins FHOD1 and PPM1F. *Molecular and Cellular Biology*.

[21] Senol O., Schaaij-Visser T. B., Erkan E. P. (2015). miR-200a-mediated suppression of non-muscle heavy chain IIb inhibits meningioma cell migration and tumor growth in vivo. *Oncogene*.

[22] Chen L., Gibbons D. L., Goswami S. (2014). Metastasis is regulated via microRNA-200/ZEB1 axis control of tumour cell PD-L1 expression and intratumoral immunosuppression. *Nature Communications*.

[23] Gigante M., Pontrelli P., Herr W. (2016). miR-29b and miR-198 overexpression in CD8+ T cells of renal cell carcinoma patients down-modulates JAK3 and MCL-1 leading to immune dysfunction. *Journal of Translational Medicine*.

[24] Liu X. F., Wang R. Q., Hu B. (2016). MiR-15a contributes abnormal immune response in myasthenia gravis by targeting CXCL10. *Clinical Immunology*.

[25] Yang H., Shao R., Huang H., Wang X., Rong Z., Lin Y. (2019). Engineering macrophages to phagocytose cancer cells by blocking the CD47/SIRP*ɑ* axis. *Cancer Medicine*.

[26] Xu S., Tao Z., Hai B. (2016). miR-424(322) reverses chemoresistance via T-cell immune response activation by blocking the PD-L1 immune checkpoint. *Nature Communications*.

[27] Xia H., Ng S. S., Jiang S. (2010). miR-200a-mediated downregulation of ZEB2 and CTNNB1 differentially inhibits nasopharyngeal carcinoma cell growth, migration and invasion. *Biochemical and Biophysical Research Communications*.

[28] Lee T. K., Cheung V. C., Lu P. (2014). Blockade of CD47-mediated cathepsin S/protease-activated receptor 2 signaling provides a therapeutic target for hepatocellular carcinoma. *Hepatology*.

[29] Zhang H., Lu H., Xiang L. (2015). HIF-1 regulates CD47 expression in breast cancer cells to promote evasion of phagocytosis and maintenance of cancer stem cells. *Proceedings of the National Academy of Sciences of the United States of America*.

[30] Kong F., Gao F., Li H. (2016). CD47: a potential immunotherapy target for eliminating cancer cells. *Clinical & Translational Oncology*.

[31] Liu J., Wang L., Zhao F. (2015). Pre-clinical development of a humanized anti-CD47 antibody with anti-cancer therapeutic potential. *PLoS One*.

