# Supplementary figures and images for: MicroRNA-200a Promotes Phagocytosis of Macrophages and Suppresses Cell Proliferation, Migration, and Invasion in Nasopharyngeal Carcinoma by Targeting CD47
Source: Biomed Res Int. 2020 Feb 20;2020:3723781. doi: 10.1155/2020/3723781 (PMC7054800; doi:10.1155/2020/3723781)

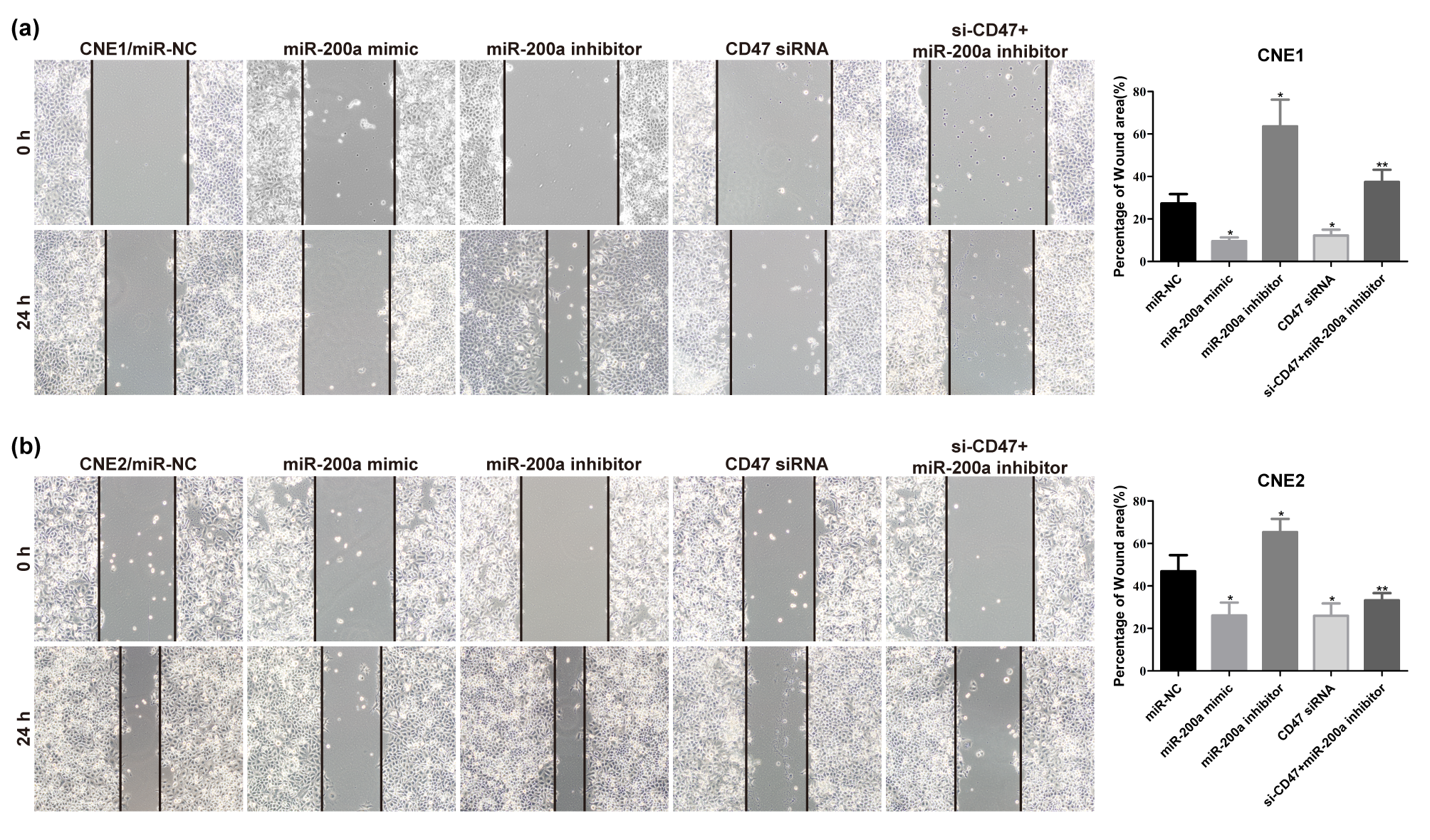

Supplement: Supplementary Materials — Supplemental Figure 1: migration of CNE1 and CNE2 cells by wound healing assay. (a) miR-200a suppresses CNE1 cell migration by downregulating CD47. (b) miR-200a suppresses CNE2 cell migration by downregulating CD47. ∗P < 0.05 vs. the miR-NC group. ∗∗P < 0.05 vs. the miR-200a inhibitor group. [file 3723781.f1.tif]
